# Independent and cumulative effects of risk factors associated with stillbirths in 50 low- and middle-income countries: A multi-country cross-sectional study

**DOI:** 10.1016/j.eclinm.2022.101706

**Published:** 2022-10-31

**Authors:** Zhihui Li, Yuhao Kong, Shaoru Chen, Maya Subramanian, Chunling Lu, Rockli Kim, Fernando C. Wehrmeister, Yi Song, S.V. Subramanian

**Affiliations:** a Vanke School of Public Health, Tsinghua University, 100084, Beijing, China; b Institute for Healthy China, Tsinghua University, 100084, Beijing, China; c Brandeis University, Waltham, MA, 02453, USA; d Department of Global Health and Social Medicine, Harvard Medical School, Boston, MA, 02115, USA; e Division of Global Health Equity, Brigham & Women's Hospital, Boston, MA, 02115, USA; f Division of Health Policy and Management, College of Health Science, Korea University, Seoul, South Korea; g Interdisciplinary Program in Precision Public Health, Department of Public Health Sciences, Graduate School of Korea University, Seoul, South Korea; h Harvard Center for Population & Development Studies, Cambridge, MA, 02115, USA; i Department of Epidemiology, Universidade Federal de Pelotas, Pelotas, Brazil; j Institute for Global Public Health, Rady Faculty of Health Sciences, University of Manitoba, Winnipeg, Canada; k Institute of Child and Adolescent Health, School of Public Health, Peking University, Beijing, 100191, China; l National Health Commission Key Laboratory of Reproductive Health, Peking University, Beijing, 100191, China; m Department of Social and Behavioral Sciences, Harvard T.H. Chan School of Public Health, Boston, MA, 02115, USA

**Keywords:** Stillbirth, Risk factors, Risk scores, Low- and middle-income countries

## Abstract

**Background:**

Early identification of high-risk pregnancies could reduce stillbirths, yet remains a challenge in low- and middle-income countries (LMICs). This study aims to estimate the associations between easily observable risk factors and stillbirths, and construct a risk score which could be adopted in LMICs to identify pregnancies with high risk of stillbirths.

**Methods:**

Using the most recent Demographic and Health Surveys from 50 low- and middle-income countries (LMICs) with available data between January 1, 2010 and December 31, 2021, we analysed a total of 22 factors associated with stillbirths in a series of single-adjusted and mutually adjusted logistic regression models. Upon identification of the risk factors with the strongest associations, we constructed a risk score on the basis of the magnitude of the β coefficient to examine the cumulative effects of risk factors on stillbirths. To assess whether the associations between risk scores and stillbirths were moderated by protective factors, we added an interaction term between the identified protective factor and risk scores to the regression model. We also conducted two sets of subgroup analyses for previous history of pregnancy and maternal age at pregnancy and four sets of supplementary analyses to test the robustness of the results.

**Findings:**

Among the 795,642 women identified for analysis with at least one pregnancy within the five years before the survey, the most recent pregnancy of 8968 (1.13%) ended as stillbirths. Using a mutually adjusted regression model, we found that the top factors showing the strongest associations with stillbirths were short maternal height (odds ratio [OR]: 1.99, 95% confidence interval [CI]: 1.48–2.67, *P* < 0.001), interpregnancy interval less than six months (OR: 1.84, 95% CI: 1.42–2.38, *P* < 0.001), previous stillbirth history (OR: 1.55, 95% CI: 1.07–2.26, *P* < 0.020), low maternal education (OR: 1.50, 95% CI: 1.01–2.24, *P* = 0.045), and lowest household wealth (OR: 1.32, 95% CI: 1.08–1.61, *P* = 0.008). A female household head was a protective factor with an OR of 0.71 (95% CI: 0.55–0.90, *P* = 0.005). Single-adjusted models, subgroup analyses, and sensitivity analyses showed generally consistent results. We also found that the odds of stillbirths increased with a larger risk score with a *P* trend <0.001. Compared with women without any risk factors, women with a risk score of 5 or more were 4.11 (95% CI: 2.83–5.97, *P* < 0.001) times more likely to have their pregnancies ending up as stillbirths. However, these associations were weakened if the head of household was female.

**Interpretation:**

Our study suggested that short maternal height, low socioeconomic status, previous stillbirth history, low maternal education, and very short interpregnancy interval had the strongest associations with stillbirths. The construction of risk scores using easily observable risk factors could be an effective way to identify high-risk pregnancies in resource-poor settings.

**Funding:**

This research was supported by Sanming Project of Medicine in Shenzhen (NO. SZSM202111001) and China National Natural Science Foundation (NO. 72203119).

**Research in context**

**Evidence before this study**

To reduce the number of stillbirths, early identification of the population at high risk is critical. We searched Google Scholar, Web of Science, and PubMed for studies with the combination of the following terms: “stillbirth,” “perinatal,” “pregnancy outcome,” and “risk factor,” “identification,” “intervention,” “prevention,” “assessment,” and “low- and middle-income countries,” with no date and language restriction, with the last search carried out June 27, 2022. Although a large number of previous studies have attempted to identify the single or a subset of risk factor(s) of stillbirth, most of them overlook the facts that the majority of families in low- and middle-income countries (LMICs) are facing the concurrence of multiple risk factors. However, there is little evidence on the cumulative effects of risk factors on stillbirth in LMICs.

**The added value of this study**

To our knowledge, this is the first global-level study to systematically assess the relative significance of factors associated with stillbirths and the cumulative effect of the concurrence of multiple risk factors. Our study identified the leading risk factors of stillbirth, including short maternal height, interpregnancy interval less than six months, previous stillbirth history, low maternal education, and lowest household wealth. Upon identification of the leading risk factors, we constructed a risk score based on the number of risk factors for each individual and their estimated magnitudes. We found that increasing risk scores were strongly associated with a rising risk of stillbirth.

**The implications of all the available evidence**

Our study suggests that prevention and intervention programmes that target the leading risk factors are needed to reduce stillbirth in LMICs, especially for families with a concurrence of multiple risk factors. More importantly, our construction of a risk score could potentially serve as a simple screening tool to identify populations with high risk of stillbirth in LMICs.

## Introduction

Stillbirth is a serious global health issue that continues to affect thousands of families every day, and has severe social, emotional, and economic consequences.^1^ The World Health Organization (WHO) defines stillbirth as a baby that is born with no signs of life at 28 weeks of gestation or later, with a birthweight of over 1000 g or a body length over 35 cm.^2^ Globally, approximately 3 million babies are stillborn per year, making the current stillbirth rate 13.9 stillbirths per 1000 total births.^2^ However, these rates are likely to be underestimates as data on stillbirth remains difficult to capture.^3^

To date, around 98% of these stillbirths are concentrated in low-to-middle income countries (LMICs), making it critical to target these specific areas for intervention and support.^4^ Nevertheless, there has been little progress on reducing stillbirths in LMICs. The “Every Newborn Action Plan” was proposed in 2014, setting an explicit goal of 12 or fewer stillbirths per 1000 births in every country worldwide by 2030.^1^ According to an estimation conducted in 2016, there were 94 of the 157 studied countries, mainly high- or upper-middle-income countries, have already met this target; while 56 countries, the majority of them being LMICs, need to make substantial progress to reach this goal.^5^ Hence, in order to accelerate the pace for achieving this challenging goal, accurate identification of populations with high risk of stillbirths is essential to guide further prevention and intervention programs.

Previous studies on LMICs have quantified the effects of risk factors on stillbirths, such as family income, household infrastructure, education, and quality of primary care.^3^^,^^5^ Most of these studies focused on a specific risk factor for stillbirth, holding everything else constant. However, families in LMICs frequently face multiple risk factors simultaneously.^6^ For example, a study on 16 sub-Saharan African countries found that around one-third of the mothers were both illiterate and suffered from child marriage.^7^ This may imply the need to examine the aggregate effects of social factors on stillbirth and identify populations with high stillbirth risks accordingly.

Risk scores have been widely adopted in clinical settings, yet mostly focus on how the concurrence of multiple biomarkers could affect a specific disease, such as cardiovascular disease (CVD), diabetes, breast cancer, etc.^8^, ^9^, ^10^ The availability of clinical and genetic information is a major constraint in LMICs; indeed, only a few clinical risk factors of stillbirths can be easily observed in LMICs, such as parental height, parental BMI, adverse prior pregnancy outcomes, etc.^11^ In 2020, Dr. Figueroa and his colleagues introduced polysocial risk scores to estimate the aggregate impact of social factors on health outcomes, which were adopted in scattered studies on cardiovascular diseases and mortality.^12^^,^^13^ Most of the social risk factors could be easily observed in LMICs, yet the inclusion of such risk factors alone might be insufficient when considering the multidimensional risks of stillbirths. In this study, we attempted to construct a risk score for stillbirths using self-reported and easily observable social and demographic risk factors in LMICs in order to identify pregnancies with high risk of stillbirths, so that targeted prevention and intervention strategies could be designed accordingly.

Using the most recent data (since 2010) from the Demographic and Health Survey (DHS), we first assess the relative importance of the observable risk factors in the context of LMICs, then construct risk scores with the risk factors most strongly associated with stillbirths, and finally estimate the cumulative influence of risk factors on stillbirth.

## Methods

### Data source and data collection

We extracted data from the Demographic and Health Surveys. There were more than 60 low- and middle-income countries (LMICs) conducted the most recent surveys between January 1, 2010 and December 31, 2021; we included 50 of the countries with available records of stillbirths. DHSs are nationally representative household surveys that use a multistage stratified clustered sampling design. In the first stage, a number of clusters are selected from a sampling frame based on a complete list of enumeration areas (EAs).^14^ A list of all the households living in each EA is generated. In the second stage, an average of 25 households is randomly selected from the household list in each EA.^14^ All household members in the households are selected to finish the survey. DHSs collect detailed information on reproductive calendar, maternal and household characteristics, pregnancy and birth outcomes, and maternal care and contraceptive use. We excluded surveys conducted before 2010 to keep the data updated, and also to avoid missing and inconsistent measurements.

We assembled data from 1,474,595 women aged 15–49 years old from 50 LMICs. The exclusion criteria for our analytic sample were as follows: (1) without pregnancy history within the five years before the survey, (2) the most recent pregnancy ended up with abortion or miscarriage, and (3) first pregnancy in process at the point of the survey. After applying the exclusion criteria, we identified a final sample of 795,642 women for our primary analyses. See Appendix Table 1 for countries included in the study.

This study was approved by the Tsinghua Institutional Review Board (Project No. 20220005). Informed consent was not required because the databases are anonymous and open to public access.

### Outcomes

The primary outcome adopted in this study was stillbirth from the most recent pregnancy. We followed the definition provided by the WHO, which considers stillbirth to be a baby who dies after 28 weeks of pregnancy before or during birth.^2^ The measure of stillbirths was generated from the DHS Contraceptive Calendar, which collects month-by-month information on women's reproductive events, such as births, pregnancies, and stillbirths. Since the DHS Contraceptive Calendar was a monthly report, we considered stillbirths to be fetal death after seven months of pregnancy. The calendar was generated based on self-reported information, but was cross-checked using birth history data collected in the DHS interview. The recorded calendar length varied somewhat from country to country, ranging between five and seven years prior to the survey. To maintain consistency across countries and to minimise self-report bias, we only considered reproductive histories up to five years preceding the survey. For our analyses, a binary variable of stillbirth was constructed with the value of 1 representing mothers with stillbirth from the most recent pregnancy, while 0 meant otherwise.

### Risk factors

Based on the previous literature summarizing the observable risk factors associated with stillbirths,^5^^,^^15^ we selected 16 factors for our primary analysis and 6 additional factors on maternal care services and paternal biological status for supplementary analysis. A detailed list and description of all risk factors can be found in Table 1.

**Table 1:** Definition of 22 factors associated with stillbirth.

Risk factors	Definition	Reference category	Category type	Self-reported
**Household characteristics**				
Household wealth	Constructed by DHS based on a selected set of household assets in 5 quintiles; 1 indicates poorest household wealth; 2, poorer; 3, middle; 4, richer; 5, richest household wealth	Richest household wealth	Categorical variable	No
Type of residence	Lives in the urban or rural, in the 2 following categories: (1) urban; (2) rural	Urban	Binary variable	No
Water source	Safe if the household had access to water piped into dwelling, yard, or plot, public tap or standpipe, tube well or borehole, protected well or spring, rain water, and bottled water; unsafe otherwise	Safe water source	Binary variable	Yes
Sanitation facility	Improved if the household had access to flush to piped sewer system, septic tank or pit latrine, ventilated improved pit latrine, pit latrine with slab, and composting toilet; unimproved otherwise	Improved sanitation facility	Binary variable	Yes
Indoor pollution	Low if the household used solid fuels for cooking; high otherwise	Low indoor pollution	Binary variable	Yes
Sex of the household head	Respondents selected the head of household if he/she usually lives in the household, main economic provider, or other reason in the 2 following categories: (1) male; (2) female.	Male	Binary variable	Yes
**Parental characteristics**				
Maternal age at the survey	The age of the respondents at the survey, in the 3 following categories: (1) 15–19 years old, (2) 20–34 years old, (3) 35–49 years old	20–34 years old	Categorical variable	Yes
Maternal age at marriage	In the 2 following categories: (1) married at <18 y and (2) married at ≥18 y, with married at <18 y defined as child marriage	Married at ≥18 y	Categorical variable	Yes
Maternal age at pregnancy	In the 3 following categories: (1) 15–19 years old, (2) 20–34 years old, (3) 35–49 years old	20–34 years old	Categorical variable	Yes
Maternal education	In the 4 following categories: (1) no schooling, (2) primary education, (3) secondary education, and (4) higher education	Higher education	Categorical variable	Yes
Paternal education	In the 4 following categories: (1) no schooling, (2) primary education, (3) secondary education, and (5) higher education	Higher education	Categorical variable	Yes
Maternal smoking	Yes if the woman smoke, no otherwise	No smoking	Binary variable	Yes
Maternal height	In the 4 following categories: (1) <145 cm, (2) 145–149.9 cm, (3) 150–159.9 cm, (4) ≥160 cm, with <145 cm defined as short maternal height	≥160 cm	Categorical variable	No
Maternal BMI	In the 3 following categories: (1) <18.5 kg/m^2^, (2) 18.5–24.9 kg/m^2^, and (3) ≥25 kg/m^2^, with <18.5 kg/m^2^ defined as low maternal BMI	≥25 kg/m^2^	Categorical variable	No
Paternal height	In the 5 following categories: (1) <160 cm, (2) 160–164.9 cm, (3) 165–169.9 cm, (4) ≥170 cm, with <160 cm defined as short paternal height	≥170 cm	Categorical variable	No
Paternal BMI	In the 3 following categories: (1) <18.5 kg/m^2^, (2) 18.5–24.9 kg/m^2^, and (3) ≥25 kg/m^2^, with <18.5 kg/m^2^ defined as low maternal BMI	≥25 kg/m^2^	Categorical variable	No
**Pregnancy history**				
Previous history of stillbirth	Yes if the mother had stillbirth history before, no otherwise	No previous history with stillbirth	Binary variable	Yes
Previous history of caesarean section	Yes if the last child was born by caesarean section, no otherwise	No previous history of caesarean section	Binary variable	Yes
Interpregnancy interval	The interval between the birth and the previously reported birth in the 9 following categories: (1) 0–5 months, (2) 6–11 months, (3) 12–17 months, (4) 18–23 months, (5) 24–35 months, (6) 36–47 months, (7) 48–59 months, (8) ≥ 60 months, (9) no previous pregnancy	18–23 months	Categorical variable	Yes
**Maternal care services received during prior pregnancy and child birth**				
Timing of the first antenatal care	Yes if the woman delivered the child with antenatal care within 12 weeks of gestation, no otherwise	Yes	Binary variable	Yes
Number of antenatal care visits	Antenatal care visits from a skilled provider for the most recent birth in the 3 following categories: (1) <4 antenatal care, (2) 4–7 antenatal care visits, and (3) ≥8 antenatal care	≥8 antenatal care	Categorical variable	Yes
Skilled birth attendants	Yes if a woman delivered the child with skilled birth attendant, including physicians, nurses, and midwives; no otherwise	With skilled birth attendant	Binary variable	Yes

Abbreviation: BMI, body mass index (calculated as weight in kilograms divided by height in meters squared).

The 22 risk factors were divided into four categories: household factors, parental factors, pregnancy history factors, and maternal care services. The six household factors were household wealth, type of residence, water source, sanitation facility, indoor pollution, and sex of the head of household. We identified a total of ten parental factors: maternal age at the survey, maternal age at marriage, maternal age at pregnancy, maternal education, paternal education, maternal smoking, maternal height, maternal body mass index (BMI, kg/m^2^), paternal height, and paternal BMI. We included three indicators of pregnancy history prior to the most recent one, which were: previous history of stillbirth, previous history of caesarean section, and interpregnancy interval. Notably, if the individual did not have a previous pregnancy, we considered them not having a previous history of stillbirth or C-section; we treated interpregnancy interval as a categorical variable and generated a separate category for those without previous pregnancy. We included three maternal care indicators, which were the timing of first antenatal care, number of antenatal care visits, and skilled birth attendants. Maternal care indicators have been addressed in a volume of previous literature.^6^ However, since these indicators were not collected for the pregnancies ending up as stillbirths in the DHS database, we used the records of antenatal and delivery care from their previous pregnancy as a proxy for the maternal care obtained for this pregnancy.

### Statistical analysis

#### Regression models

We pooled data from all countries to assess the association between the risk factors and stillbirth. We included the sampling weight, clustering, and stratification variables provided by the DHS to ensure our estimates were representative in both pooled and national-level analyses.^16^ Our sample was then clustered at the level of the primary sampling unit, allowing for interdependence of error terms within clusters and households.^16^ In pooled analyses, we reweighted observations to account for the country's population size as done previously,^17^ and used country fixed effects by adding a dichotomous variable for each country to account for the unobservable country-level factors.

We developed two sets of logistic regression models: first, we performed a single-adjusted model for each risk factor adjusting for maternal age at the survey. Second, we ran a mutually adjusted model in which we simultaneously included all of the risk factors, as well as adjusting maternal age at the survey. Prior to multivariate analysis, we examined the multicollinearity using variance inflation factor (VIF) (Appendix Table 2). We found that no variable had a high VIF value. For all risk factors, we followed previous practice^17^ and used the best-off group as the reference group to ensure consistency in interpretation of odds ratios.^18^ For factors with multiple categories (e.g., household wealth quintiles), our results presented the ORs that corresponded to the worst-off group (e.g., the poorest quintile). The factors with ORs significantly greater than one were identified as risk factors of stillbirths; those with ORs significantly less than one were identified as protective factors.

#### Risk score construction and the identification of cumulative effects

For each individual, we constructed a risk score to measure how the concurrence of risk factors was related to stillbirths with the following four steps:

Firstly, following previous practice,^17^ we compared and ordered the coefficient sizes of the risk factors based on the regression results from the mutually adjusted models. The risk factors with significant effect sizes were identified as the leading factors associated with stillbirth.

Secondly, we derived a scoring system based on the results of the mutually adjusted model. Score was assigned to each of the top five significant variables on the basis of the magnitude of the β coefficient. For each individual, we calculated a total score for the risk of stillbirths by summing up the scores for all five variables.

Thirdly, we investigated the associations between risk scores and stillbirths using logistic regression adjusting for maternal age at the survey and number of children in the household. Similarly, as before, we included sample weight, country size, and country fixed effects in the regression. Moreover, to assess whether the associations between risk factors and stillbirths were affected by protective factors, we added an interaction term between the identified protective factor and risk scores.

Lastly, we calculated the sensitivity, specificity, positive predictive value (PPV), and negative predictive value (NPV) for several cut-off scores; following previous practice,^19^ we took the cut-off score with the maximum sum of sensitivity and specificity as the optimum.

#### Subgroup and sensitivity analyses

We generated two sets of subgroup analyses according to previous history of pregnancy (with or without previous pregnancies) and maternal age at pregnancy (15–19, 20–34, and 35–49 years old). The subgroups by history of pregnancy aimed to distinguish primipara and multipara. Previous evidence showed that the risk factors and their relative importance might potentially differ between primipara and multipara; for multipara, previous pregnancy outcome might be an important risk factor for stillbirths, which could not be observed among primipara.^20^ Various maternal ages at pregnancy might be linked with risk of stillbirths in different ways – adolescent pregnancies were more prone to occur with lower socioeconomic conditions, while pregnancy at older ages might be more related to declined fertility and placenta function.^21^ Therefore, we stratified the sample by maternal age at pregnancy to investigate the relative importance of risk factors in each subgroup.

Meanwhile, we developed four sets of supplementary analyses: first, we included three risk factors of maternal care services from the pregnancy prior to the latest one in the mutually adjusted model. Although maternal care services were closely related to stillbirths, we lacked such information for women with pregnancies ending up as stillbirths. Therefore, we generated a sub-sample of 272,129 women who had at least one live birth prior to the last pregnancy and adopted the maternal care services reported for the live birth as a proxy for the latest pregnancy.

Second, we added three risk factors of paternal indicators to the mutually adjusted model for a subset of 54,248 observations that had collected information on fathers. Growing literature indicates that paternal biological characteristics play a critical role in pregnancy outcomes,^22^ yet the data collection process on the related indicators lags behind – among the 50 countries, only 13 of them collected paternal indicators. Therefore, we did not include them in the primary analysis.

Third, we included maternal BMI at the survey as a proxy of the women's general weight status.

Last, to further simplify the identification of high-risk pregnancies for national and local healthcare providers, we constructed another set of risk scores by counting the number of risk factors. To differentiate them with the risk scores accounting for the regression coefficients, we named them unweight risk score. For example, if an individual simultaneously had two of the five identified risk factors, she would be assigned a risk score of 2. In practice, considering the small number of individuals with four or five risk factors, we combined these two categories during our analyses. Consequently, the risk scores using this method were on a scale of 0–4. Similar as above, we examined the associations between risk scores and stillbirths with logistic regression adjusting for maternal age at the survey, number of children in the household, and country-fixed effects.

All analyses were conducted using Stata 17.0. For observations with missing data on one or more risk factors, the MI commands were conducted for multiple imputations. Following previous practice, we assumed that the data were missing at random.^23^^,^^24^ We performed multiple imputation by chained equations and logistic regression in the pooled dataset with 795,642 observations from 50 LMICs. In the pooled dataset, 337,965 (42.4%) had missing information for at least one of the 22 risk factors. Given the considerably high proportion of missing cases in our data, we imputed 10 datasets. The risk factors with missing information were imputed based on country, wealth index, place of residence, maternal age at the survey, current marital status, number of children in the household, and sex of the household head. All statistical tests were 2-tailed, and *P* < 0.05 was considered statistically significant.

### Role of the funding source

The funders had no role in the design and conduct of the study, nor the decision to prepare and submit the manuscript for publication. ZL, YK, YS, and SV had full access to all of the data in the study. All authors had final responsibility for the decision to submit for publication.

## Results

A total of 795,642 women aged 15–49 from 50 LMICs with at least one pregnancy were identified for analyses, including 8968 with previous stillbirth history and 786,674 without previous stillbirths. Overall, a total of 198,224 (24.91%) women were from the poorest quintile households and 554,901 (69.74%) lived in rural areas. Compared with mothers who had a live birth for the last pregnancy, we found mothers who had a stillbirth were more likely to be poorer, from rural area, with lower parental education, without improved sanitation facilities, suffering from indoor pollution, and living with a male head of household. They were also more likely to get married at a young age, with shorter height, and with a stillbirth prior to the most recent pregnancy (Table 2 and Appendix Table 3).

**Table 2:** Summary table of the sample characteristics.

	All women	The most recent pregnancy ended up with a stillbirth	The most recent pregnancy ended up with a live birth	*P* value
Total number of observations	795,642	8968	786,674	
**Household characteristics**				
**Wealth quintile**				<0.001
Poorest	198,224 (24.91%)	2418 (26.96%)	195,806 (24.89%)	
Poorer	177,470 (22.31%)	2157 (24.05%)	175,313 (22.29%)	
Middle	158,189 (19.88%)	1783 (19.88%)	156,406 (19.88%)	
Richer	141,576 (17.79%)	1518 (16.93%)	140,058 (17.80%)	
Richest	120,183 (15.11%)	1092 (12.18%)	119,091 (15.14%)	
**Type of residence**				<0.001
Urban	240,741 (30.26%)	2280 (25.42%)	238,461 (30.31%)	
Rural	554,901 (69.74%)	6688 (74.58%)	548,213 (69.69%)	
**Safe water**				<0.001
Yes	616,879 (77.53%)	6765 (75.43%)	610,114 (77.56%)	
No	169,342 (21.28%)	2165 (24.14%)	167,177 (21.25%)	
Missing	9421 (1.18%)	38 (0.42%)	9383 (1.19%)	
**Improved sanitation facility**				<0.001
No	306,618 (38.54%)	3937 (43.90%)	302,681 (38.48%)	
Yes	484,259 (60.86%)	4976 (55.49%)	479,283 (60.93%)	
Missing	4765 (0.60%)	55 (0.61%)	4710 (0.60%)	
**Low indoor pollution**				<0.001
Yes	245,309 (30.83%)	2123 (23.67%)	243,186 (30.91%)	
No	503,443 (63.28%)	6405 (71.42%)	497,038 (63.18%)	
Missing	46,890 (5.89%)	440 (4.91%)	46,450 (5.90%)	
**Sex of the household head**				0.037
Male	661,632 (83.16%)	7531 (83.98%)	654,101 (83.15%)	
Female	134,010 (16.84%)	1437 (16.02%)	132,573 (16.85%)	
**Parental characteristics**				
**Maternal age at the survey**				<0.001
15–19	35,549 (4.47%)	508 (5.66%)	35,041 (4.45%)	
20–34	610,377 (76.72%)	6346 (70.76%)	604,031 (76.78%)	
35–49	149,716 (18.82%)	2114 (23.57%)	147,602 (18.76%)	
**Maternal age at marriage**				<0.001
<18 years old	302,216 (37.98%)	3783 (42.18%)	298,433 (37.94%)	
≥18 years old	470,597 (59.15%)	4944 (55.13%)	465,653 (59.19%)	
Missing	22,829 (2.87%)	241 (2.69%)	22,588 (2.87%)	
**Maternal age at last pregnancy**				<0.001
15–19	138,977 (17.47%)	1725 (19.24%)	137,252 (17.45%)	
20–34	578,555 (72.72%)	5975 (66.63%)	572,580 (72.78%)	
35–49	78,110 (9.82%)	1268 (14.14%)	76,842 (9.77%)	
**Maternal education**				<0.001
No education	233,529 (29.35%)	3195 (35.63%)	230,334 (29.28%)	
Primary education	185,354 (23.30%)	2226 (24.82%)	183,128 (23.28%)	
Secondary education	281,357 (35.36%)	2651 (29.56%)	278,706 (35.43%)	
Higher education	77,107 (9.69%)	640 (7.14%)	76,467 (9.72%)	
Missing	18,295 (2.30%)	256 (2.85%)	18,039 (2.29%)	
**Paternal education**				<0.001
No education	144,502 (18.16%)	2044 (22.79%)	142,458 (18.11%)	
Primary education	132,044 (16.60%)	1668 (18.60%)	130,376 (16.57%)	
Secondary education	168,496 (21.18%)	1635 (18.23%)	166,861 (21.21%)	
Higher education	52,300 (6.57%)	502 (5.60%)	51,798 (6.58%)	
Missing	298,300 (37.49%)	3119 (34.78%)	295,181 (37.52%)	
**Maternal smoking**				<0.001
Yes	687,861 (86.45%)	7886 (87.93%)	679,975 (86.44%)	
No	9592 (1.21%)	96 (1.07%)	9496 (1.21%)	
Missing	98,189 (12.34%)	986 (10.99%)	97,203 (12.36%)	
**Maternal height**				<0.001
<145 cm	42,246 (5.31%)	679 (7.57%)	41,567 (5.28%)	
145–149.9 cm	99,277 (12.48%)	1119 (12.48%)	98,158 (12.48%)	
150–159.9 cm	284,168 (35.72%)	2918 (32.54%)	281,250 (35.75%)	
≥160 cm	111,046 (13.96%)	948 (10.57%)	110,098 (14.00%)	
Missing	258,905 (32.54%)	3304 (36.84%)	255,601 (32.49%)	
**Maternal BMI**				<0.001
<18.5 kg/m^2^	70,165 (8.82%)	696 (7.76%)	69,469 (8.83%)	
18.5–25 kg/m^2^ (not include 25 kg/m^2^)	321,665 (40.43%)	3340 (37.24%)	318,325 (40.46%)	
25 kg/m^2^ or more	144,273 (18.13%)	1619 (18.05%)	142,654 (18.13%)	
Missing	259,539 (32.62%)	3313 (36.94%)	256,226 (32.57%)	
**Pregnancy history**				
**Had a stillbirth prior to the most recent pregnancy**				<0.001
Yes	6687 (0.84%)	270 (3.01%)	6417 (0.82%)	
No	534,721 (67.21%)	6182 (68.93%)	528,539 (67.19%)	
Missing	254,234 (31.95%)	2516 (28.06%)	251,718 (32.00%)	
**Had a caesarean section prior to the most recent pregnancy**				<0.001
Yes	97,302 (12.23%)	822 (9.17%)	96,480 (12.26%)	
No	671,772 (84.43%)	5647 (62.97%)	666,125 (84.68%)	
Missing	26,568 (3.34%)	2499 (27.87%)	24,069 (3.06%)	
**Interpregnancy interval**				<0.001
0–5	44,001 (5.53%)	617 (6.88%)	43,384 (5.51%)	
6–11	46,289 (5.82%)	565 (6.30%)	45,724 (5.81%)	
12–17	79,947 (10.05%)	829 (9.24%)	79,118 (10.06%)	
18–23	76,094 (9.56%)	799 (8.91%)	75,295 (9.57%)	
24–35	122,527 (15.40%)	1149 (12.81%)	121,378 (15.43%)	
36–47	75,379 (9.47%)	694 (7.74%)	74,685 (9.49%)	
48–59	45,604 (5.73%)	436 (4.86%)	45,168 (5.74%)	
60 or more	87,931 (11.05%)	1095 (12.21%)	86,836 (11.04%)	
No previous pregnancy	217,870 (27.38%)	2784 (31.04%)	215,086 (27.34%)	

### Pooled analyses

**Fig. 1:**
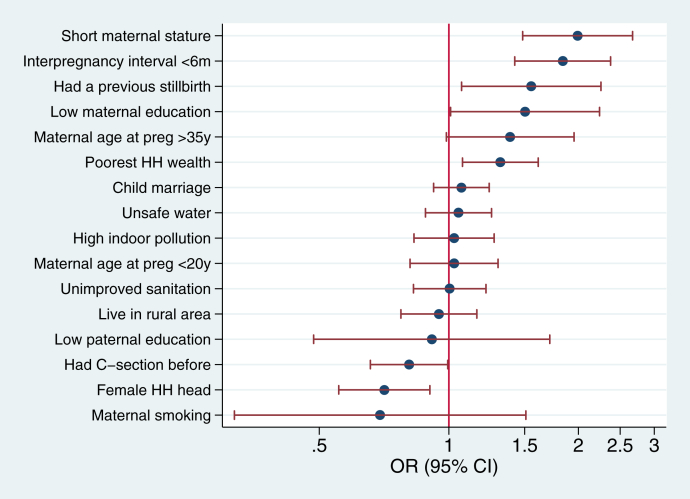
**Relative ranking of risk factors associated with stillbirths from mutually adjusted model in pooled analysis of 50 countries, odds ratio (ORs) and 95% confidence interval (CI)**. Low maternal/paternal education – mothers/fathers with no education; short maternal stature – maternal height of less than 145 cm; child marriage – mother younger than 18 years at marriage; poorest HH wealth – household with the poorest wealth status; high indoor pollution – household not using solid fuels for cooking; C-section – caesarean section ∗Maternal age at the survey was adjusted in this model.

We present the results of the pooled analyses in Fig. 1 (mutually adjusted model) and Appendix Fig. 1 (single-adjusted model). In the single-adjusted regression models, we found that 13 of the 16 influencing factors were significantly associated with a higher likelihood of stillbirth. Among them, low maternal education had the strongest association with stillbirth (OR: 3.63, 95% CI: 3.23–4.09, *P* < 0.001), followed by having a stillbirth prior to the most recent pregnancy (OR: 3.22, 95% CI: 2.56–4.05, *P* < 0.001), short maternal height (OR: 2.73, 95% CI: 2.37–3.13, *P* < 0.001), and poorest household wealth (OR: 2.17, 95% CI: 1.96–2.41, *P* < 0.001). There were two influencing factors associated with lower odds of stillbirths, which were with female head of household (OR: 0.85, 95% CI: 0.79–0.92, *P* < 0.001) and having a caesarean section prior to the most recent pregnancy (OR: 0.87, 95% CI: 0.79–0.97, *P* < 0.001).

When using the mutually adjusted models, the magnitudes of most factors attenuated substantially; yet 5 of the 16 influencing factors remained significantly positive when holding the other risk factors constant (Fig. 1). They were short maternal height (OR: 1.99, 95% CI: 1.48–2.67, *P* < 0.001), interpregnancy interval less than six months (OR: 1.84, 95% CI: 1.42–2.38, *P* < 0.001), having a previous stillbirth prior to the most recent pregnancy (OR: 1.55, 95% CI: 1.07–2.26, *P* < 0.020), low maternal education (OR: 1.50, 95% CI: 1.01–2.24, *P* = 0.045), and lowest household wealth (OR: 1.32, 95% CI: 1.08–1.61, *P* = 0.008). As in the single-adjusted models, female head of household and had caesarean section before were protective factors which were negatively associated with stillbirths, with ORs of 0.71 (95% CI: 0.55–0.90, *P* = 0.005) and 0.81 (95% CI: 0.66–0.99, *P* = 0.044), respectively.

The results of subgroup analyses by previous history of pregnancy and maternal age at pregnancy are presented in Appendix Figs. 2 and 3. The other top risk factors in various subgroups were generally consistent as the overall analyses.

### Construction of risk scores and the associations with stillbirths

Upon identification of the top significant risk factors using the mutually adjusted models (i.e. short maternal height, interpregnancy interval less than six months, previous stillbirth prior to the most recent pregnancy, low maternal education, and lowest household wealth), we constructed risk scores with these risk factors and weighted them with the estimated coefficients. Among the 795,642 women included in the study, 8968 (1.12%) had the most recent pregnancy ending up with a stillbirth. As the risk score increased, the percentage of stillbirths grew from 0.97% among those with a risk score of 0 (not presenting any of the five risk factors) to 3.15% among those with a risk score of 5 or more (see Appendix Table 4).

We presented the associations between risk scores and stillbirths in Table 3. The results showed a stepwise increase in the ORs for each additional risk factor. Specifically, compared with women with a risk score of 0, women with a score of 1–2 had 46% higher odds of ending up with stillbirths (OR: 1.46, 95% CI: 1.38–1.56, *P* < 0.001). There was an incremental increase of stillbirths as the risk score increased with a *P* trend <0.001. When the women had a risk score of 5 or more, their pregnancies had an OR of 4.11 (95% CI: 2.83–5.97, *P* < 0.001) to end up as stillbirths compared with their peers without any risk factors.

**Table 3:** The associations between risk scores and stillbirths by maternal age at last pregnancy, odds ratio (ORs) and 95% confidence interval (CI).^a^^,^^b^

Risk scores	All women	Maternal age at last pregnancy		
		15–19	20–34	35–49
0	Ref	Ref	Ref	Ref
1-2, not including 2	1.46 (1.38, 1.56)	1.44 (1.26, 1.64)	1.52 (1.41, 1.65)	1.21 (1.01, 1.44)
2-3, not including 3	1.77 (1.58, 1.99)	1.71 (1.35, 2.17)	1.94 (1.69, 2.22)	1.28 (1.00, 1.64)
3-4, not including 4	2.45 (2.18, 2.76)	2.10 (1.34, 3.26)	2.49 (2.15, 2.89)	2.22 (1.46, 3.35)
4-5, not including 5	3.23 (2.75, 3.80)	2.58 (2.00, 3.34)	3.86 (3.21, 4.64)	2.27 (1.47, 3.50)
5 or more	4.11 (2.83, 5.97)	3.37 (2.89, 3.93)	4.77 (3.15, 7.22)	3.40 (2.06, 6.94)
P trend	<0.001	<0.001	<0.001	<0.001

a The risk factors adopted in the construction of risk scores include short maternal height (<145 cm), interpregnancy interval less than six months, previous stillbirth prior to the most recent pregnancy, low maternal education (no education), and low household wealth (poorest wealth quintile).

b Risk score was calculated using the predicted value based on the top five risk factors, accounting for the estimated coefficients from the mutually adjusted model.

**Fig. 2:**
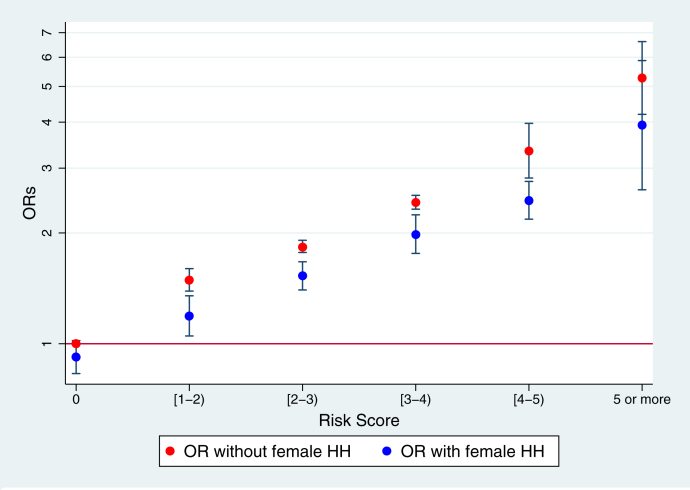
**The interaction between risk scores and stillbirths by household head (HH), odds ratio (ORs) and 95% confidence interval (CI)** 1. The risk factors adopted in the construction of risk scores include short maternal height (<145 cm), interpregnancy interval less than six months, previous stillbirth prior to the most recent pregnancy, low maternal education (no education), and low household wealth (poorest wealth quintile). 2. Risk score was calculated using the predicted value based on the top five risk factors, accounting for the estimated coefficients from the mutually adjusted model. 3. The symbol of “[” suggests “including” and the symbol of “)” suggests “not including”. For example, “[1–2)” means the risk score to be between 1 and 2, in which 2 is not included.

As female head of household was identified as a strong protective factor for stillbirths in pooled analyses, we assessed whether it would affect the associations between the risk scores and stillbirths with an interaction term between female head of household and risk factors (Fig. 2). We found that the effect of risk scores on stillbirth was moderated by sex of the head of household. A female head of household significantly weakened the associations between risk scores and stillbirths at all different levels of risk. For example, when the risk score is between 3 and 4, the ORs of stillbirths for women in a household with a male head of household was 2.42 (95% CI: 2.32, 2.53, *P* < 0.001), which is significantly higher than those with female head of household (OR: 1.98, 95% CI: 1.76, 2.24). With a cut-off score of ≥2, the sensitivity, which presented the ability of the risk score to identify high-risk pregnancies when present, reached 48%; meanwhile, the specificity, which presented the ability of the risk score to identity low-risk pregnancies when present, was 85% (Appendix Table 5).

### Sensitivity analyses

We conducted four sets of sensitivity analyses as follows: in the first supplementary analysis, we included three additional indicators of maternal care. We found that none of the three factors showed a significant association. Furthermore, as in the mutually adjusted model, we found previous stillbirth prior to the most recent pregnancy, low maternal education, and short maternal height were still the top three leading risk factors. Although the ranks of lowest household wealth and mothers younger than 18 years at marriage dropped by one, they still ranked as the top five and six risk factors (Appendix Fig. 4). In the second and third sensitivity analyses, we added paternal indicators and maternal weight indicators, and our results remained very consistent (Appendix Figs. 5 and 6). In the fourth sensitivity analysis, we generated the unweighted risk score for each individual by counting the number of the risk factors. The results were similar as using the risk scores accounting for the regression magnitudes (Appendix Table 6).

## Discussion

Our study used nationally representative data from 50 LMICs and has produced three salient findings from our analyses. First, in our pooled analyses, we identified five leading risk factors of stillbirth, including short maternal height, interpregnancy interval less than six months, previous stillbirth prior to the most recent pregnancy, low maternal education, and lowest household wealth. This finding was largely consistent in the primary, subgroup, and supplementary analyses. Second, our estimates of the cumulative effects indicated that increased risk scores were significantly associated with incremental risks of stillbirth. Third, we found female head of household to be a significant protective factor even after covariates adjustment. Moreover, we found a female head of household could effectively reduce the risks of stillbirths at different levels of risk scores.

Our study showed that short maternal height (<145 cm) and short interpregnancy interval to be strongly related to stillbirths. This finding was supported by previous evidence showing that shorter maternal height to be associated with adverse pregnancy and birth outcome, such as preterm birth, low birthweight, child mortality, low chance of spontaneous onset of labour, and high frequency of birth asphyxia.^25^^,^^26^ A couple of studies have also identified a strong association between short interpregnancy interval and stillbirth, which might possibly be attributed to nutritional depletion and related body mass reduction and micronutrient deficiency.^27^

Moreover, our study showed that previous stillbirth history to be strongly associated with the recurrence of subsequent stillbirth. This finding was consistent with considerable previous research,^6^ which showed that mothers with prior stillbirths present a 2–10 times higher possibility of subsequent stillbirth.^28^^,^^29^ This association might be attributed to the increased possibility of placental abruption, preeclampsia, preterm infant, ruptured uterus, and miscarriage caused by previous maternal complications.^30^^,^^31^ Additionally, restricted medical resources, toxic environmental conditions, and poor nutritional supplies in LMICs, which may have influenced prior pregnancy outcomes, were hard to modify in a short time, and were likely to affect the subsequent pregnancy constantly.^32^

We found female head of household to be a protective factor in stillbirth. Female-headed households were traditionally considered a vulnerable population in society. However, a growing amount of literature has shown that female household heads by choice and married female household heads tended to be more economically and socially empowered.^33^ Numerous studies have confirmed that more empowered women appeared to have better utilization of maternal care services, lower maternal mortality, and better pregnancy outcomes.^34^^,^^35^ Studies have indicated that families in LMICs with female household heads were associated with improved childcare, nutrition status, and child health.^36^ Nevertheless, some studies were unable to verify this association.^37^ Our results of an interaction between female head of household and other modifiable factors might be an indicator for researchers and policymakers to explore women empowerment in practice.

Unlike previous studies that developed conventional risk assessment tools based on clinical biomarkers and genetic variations,^38^ our study attempted to develop a set of risk scores that focused on the most observable risk factors. Although clinical assessment guidelines have been widely adopted in developed countries to identify high-risk pregnancies, most LMICs might fall short of the basic criteria to fulfil clinical applicability.^39^ Despite broad discussions on the relationship between poor socioeconomic status and unfavourable health outcomes, social factors were rarely involved in the construction of risk scores in practice. A recent study on US residents adopted the concept of polysocial risk score using seven indicators (e.g., unemployment, low income, food insecurity, lack of access to high school education, etc.) and found the ones with the highest risk scores to have 4-folder higher prevalence of atherosclerotic cardiovascular disease than those with the lowest risk scores.^40^ The risk score we developed in this study could possibly serve as a simple screening tool to identify high-risk pregnancy based on observable risk factors in LMICs. At the cut-off score of 2, the sensitivity of our scoring system reached a sensitivity of 48% and a specificity of 85%, which presents a high potential to serve as a screening tool in the resource-limited settings.

There were several limitations in our study. First, only 50 LMICs with available data were included in the present study; consequently, we were unable to generalise our results at a global level or to other income groups. Second, the usage of observational data and cross-sectional analyses hampered our ability to make any causal inference. Third, our study might confront confounding problems since the DHS did not collect information (e.g., the amount of antenatal care, timing of antenatal care, etc.) for those pregnancies which turned out to be stillbirths. Although we conducted sensitivity analyses using the maternal care received from previous pregnancies as proxies for the most recent one, we could only limit our analyses to the subsample with at least one previous live birth. Moreover, we were unable to include all the observable risk factors or the biomarkers during pregnancy in our analyses, such as medical history, pregnancy complications, blood glucose, blood pressure, haemoglobin level, etc. Fourth, given the self-report characteristic of the DHS dataset, some factors in our analyses might suffer from measurement and misreporting errors. Last but not least, due to the sample size issue, we didn't assess cross-country heterogeneity in our study, which deserved further investigation.

Using the most comprehensive and up-to-date data from 50 LMICs, our findings determined that mothers with short maternal height, interpregnancy interval less than six months, previous stillbirth history, low maternal education, and poor household wealth are the vulnerable and marginal groups with a high risk of stillbirth. Our results on the cumulative effects suggested that the development of risk scores using the most observable risk factors might serve as a potentially beneficial tool to identify high-risk pregnancies.

## Contributors

ZL, YK, YS, and SV had full access to all of the data in the study and take responsibility for the integrity of the data and the accuracy of the data analysis. ZL, YK, SC, MS, CL, RK, YS, FW, and SV had verified the data. ZL, YK, YS, FW, and SV conceptualised and designed the study. ZL led the data analysis and interpretation. ZL wrote the initial manuscript. ZL, YK, SC, MS, CL, RK, YS, FW, and SV contributed to the data analysis, interpretation of the results, and the writing. All authors contributed to the critical revision of the manuscript for important intellectual content. ZL, YS, and SV provided overall supervision of the study. All of the authors approved the final submission of the study.

## Data sharing statement

The data used in the analyses is publicly available upon the request from DHS. More information on DHS can be found at https://dhsprogram.com/, where survey datasets can be obtained.

## Declaration of interests

The authors declare no competing interests.
