# Supplementary material for: Independent and cumulative effects of risk factors associated with stillbirths in 50 low- and middle-income countries: A multi-country cross-sectional study
Source: eClinicalMedicine. 2022 Oct 31;54:101706. doi: 10.1016/j.eclinm.2022.101706 (PMC9637680; doi:10.1016/j.eclinm.2022.101706)
Supplement: Supplementary data [file mmc1.pdf]

Appendix

Appendix Table 1. Countries included in the study
Appendix Table 2. Variance inflation factor (VIF) for the risk factors
Appendix Table 3. Summary table of paternal biological and maternal care indicators
Appendix Table 4. Chances of stillbirths by risk scores
Appendix Table 5. Sensitivity, specificity, positive predictive value (PPV), and negative predictive value (NPV) of each cut-off value for the risk score
Appendix Table 6. The associations between unweighted risk scores and stillbirths
Appendix Figure 1. Relative ranking of risk factors associated with stillbirths from single-adjusted models, pooled analysis of 50 countries (N=795,642)
Appendix Figure 2. Relative ranking of factors associated with stillbirths from mutually-adjusted models, stratified by previous pregnancy history
Appendix Figure 3. Relative ranking of risk factors associated with stillbirths from mutually-adjusted models, stratified by maternal age at pregnancy
Appendix Figure 4. Relative ranking of risk factors associated with stillbirths from mutually-adjusted models, adding maternal care indicators (N=272,129)
Appendix Figure 5. Relative ranking of risk factors associated with stillbirths from mutually-adjusted models, adding paternal indicators (N=54,248)
Appendix Figure 6. Relative ranking of risk factors associated with stillbirths from mutually-adjusted models, adding maternal weight indicators (N=795,642)

Appendix Table 1. Countries included in the study

Country	Year	Number of observations
Afghanistan	2015	35,724
Albania	2017-18	2,954
Angola	2015-16	14,846
Armenia	2015-16	2,573
Bangladesh	2017-18	10,042
Benin	2017-18	14,223
Burkina Faso	2010	15,613
Burundi	2016-17	14,016
Cambodia	2014	9,311
Colombia	2015	13,652
Comoros	2012	3,376
Egypt	2014	17,800
Ethiopia	2016	11,072
Gambia	2019-20	9,175
Ghana	2014	6,778
Guatemala	2014-15	13,368
Guinea	2018	8,417
Honduras	2011-12	11,938
India	2019-21	256,998
Indonesia	2017	19,924
Jordan	2017-18	11,955
Kenya	2014	10,613
Kyrgyz Republic	2012	5,561
Lesotho	2014	3,354
Liberia	2019-20	6,258
Malawi	2015-16	17,783
Maldives	2016-17	3,538
Mali	2018	10,309
Mauritania	2019-2021	12,286

Mozambique	2011	11,675
Myanmar	2015-16	5,271
Namibia	2013	5,301
Nepal	2016	6,228
Niger	2012	35,846
Nigeria	2018	35,846
Pakistan	2017-18	14,933
Papua New Guinea	2016-18	9,628
Peru	2012	8,879
Rwanda	2020	8,746
Senegal	2019	6,577
Sierra Leone	2019	10,252
South Africa	2016	3,804
Tajikistan	2017	7,250
Tanzania	2015-16	11,039
Timor-Leste	2016	7,364
Turkey	2013	4,429
Uganda	2016	16,920
Yemen	2013	18,253
Zambia	2018	10,318
Zimbabwe	2015	6,514

Appendix Table 2. Variance inflation factor (VIF) for the risk factors

	**VIF**
Child marriage	1.47
Female household head	1.04
Had a previous stillbirth	1.02
Had C-section before	1.06
High indoor pollution	2.03
Interpregnancy interval <6m	1.24
Live in rural area	1.51
Low maternal education	2.36
Low paternal education	2.07
Maternal age at preg <20y	1.45
Maternal age at preg >35y	2.14
Maternal smoking	1.27
Poorest household wealth	2.13
Short maternal stature	1.16
Unimproved sanitation	1.68
Unsafe water	1.26

Appendix Table 3. Summary table of paternal biological and maternal care indicators

	All women	The most recent pregnancy ended up with a stillbirth	The most recent pregnancy ended up with a live birth	p value
		Parental characteristics (*N=54,248*)		
**Paternal height**				0.626
<160cm	12,430 (22.91%)	125 (23.36%)	12,305 (22.91%)	
160-164.9cm	12,903 (23.79%)	131 (24.49%)	12,772 (23.78%)	
165-169.9cm	13,485 (24,86%)	120 (22.43%)	13,365 (24.88%)	
≥170cm	15,430 (28.44%)	159 (29.72%)	15,271 (28.43%)	
**Paternal BMI**				0.521
<18.5 kg/m^2	6,465 (11.92%)	66 (12.34%)	6,399 (11.91%)	
18.5-25 kg/m^2 (not include 25 kg/m^2)	36,815 (67.86%)	371 (69.35%)	36,444 (67.85%)	
25 kg/m^2 or more	10,907 (20.11%)	97 (18.13%)	10,810 (20.13%)	
Missing	61 (0.11%)	1 (0.19%)	60 (0.11%)	
		Maternal care received during prior pregnancy and child birth (N=272,129)		
**Timing of the first antenatal visit**				<0.001
Yes	117,208 (43.07%)	1,195 (40.20%)	116,013 (43.10%)	
No	111,131 (40.84%)	1,230 (41.37%)	109,901 (40.83%)	
Missing	43,790 (16.09%)	548 (18.43%)	43,242 (16.07%)	
**Antenatal care for the pregnancy prior to the most recent pregnancy**				0.002
<4	136,219 (50.06%)	1,559 (52.44%)	134,660 (50.03%)	
4-7	94,602 (34.76%)	1,011 (34.01%)	93,591 (34.77%)	
8 or more	19,393 (7.13%)	154 (5.18%)	19,239 (7.15%)	
Missing	21,915 (8.05%)	249 (8.38%)	21,666 (8.05%)	
**Skilled birth attendant for the pregnancy prior the most recent pregnancy**				<0.001
Yes	167,604 (61.59%)	1,714 (57.65%)	165,890 (61.63%)	
No	92,645 (34.04%)	1,114 (37.47%)	91,531 (34.01%)	
Missing	11,880 (4.37%)	145 (4.88%)	11,735 (4.36%)	

Appendix Table 4. Chances of stillbirths by risk scores[1,2]

Risk scores	All women	Women with the most recent pregnancy ended up with a stillbirth	Percentage (%)
**Total**	795,642	8,968	1.13
**0**	406,498	3,950	0.97
**1-2, not including 2**	268,053	3,196	1.19
**2-3, not including 3**	78,013	983	1.26
**3-4, not including 4**	29,333	518	1.77
**4-5, not including 5**	12,158	271	2.23
**5 or more**	1,587	50	3.15

Note:

1. The risk factors adopted in the construction of risk scores include short maternal height (<145cm), interpregnancy interval less than six months, previous stillbirth prior to the most recent pregnancy, low maternal education (no education), and low household wealth (poorest wealth quintile).
2. Risk score was calculated using the predicted value based on the top five risk factors, accounting for the estimated coefficients from the mutually adjusted model.

Appendix Table 5. Sensitivity, specificity, positive predictive value (PPV), and negative predictive value (NPV) of each cut-off value for the risk score

Cutpoint	Sensitivity	Specificity	PPV	NPV
**>=1**	75.60	51.61	2.00	99.39
**>=2**	48.01	85.07	4.68	99.08
**>=3**	36.03	94.82	12.14	98.68
**>=4**	17.49	98.36	20.32	98.03
**>=5**	6.51	99.82	54.06	97.04

Note:

1. The risk factors adopted in the construction of risk scores include short maternal height (<145cm), interpregnancy interval less than six months, previous stillbirth prior to the most recent pregnancy, low maternal education (no education), and low household wealth (poorest wealth quintile).
2. Risk score was calculated using the predicted value based on the top five risk factors, accounting for the estimated coefficients from the mutually adjusted model.

Appendix Table 6. The associations between unweighted risk scores and stillbirths

Risk scores	All women	Women with the most recent pregnancy ended up with a stillbirth	Percentage (%)	Odds ratio (95% confidence interval)
**0**	406,498	3,950	0.97	Ref
**1**	268,053	3,196	1.19	1.47 (1.38, 1.56)
**2**	78,013	983	1.26	1.98 (1.80, 2.17)
**3**	29,333	518	1.77	3.24 (2.78, 3.80)
**4**	12,158	271	2.23	4.91 (2.94, 8.21)

1. The risk factors adopted in the construction of risk scores include short maternal height (<145cm), interpregnancy interval less than six months, previous stillbirth prior to the most recent pregnancy, low maternal education (no education), and low household wealth (poorest wealth quintile).
2. Risk score was calculated by counting the number of the risk factors.

Appendix Figure 1. Relative ranking of risk factors associated with stillbirths from single-adjusted models, pooled analysis of 50 countries (N=795,642)

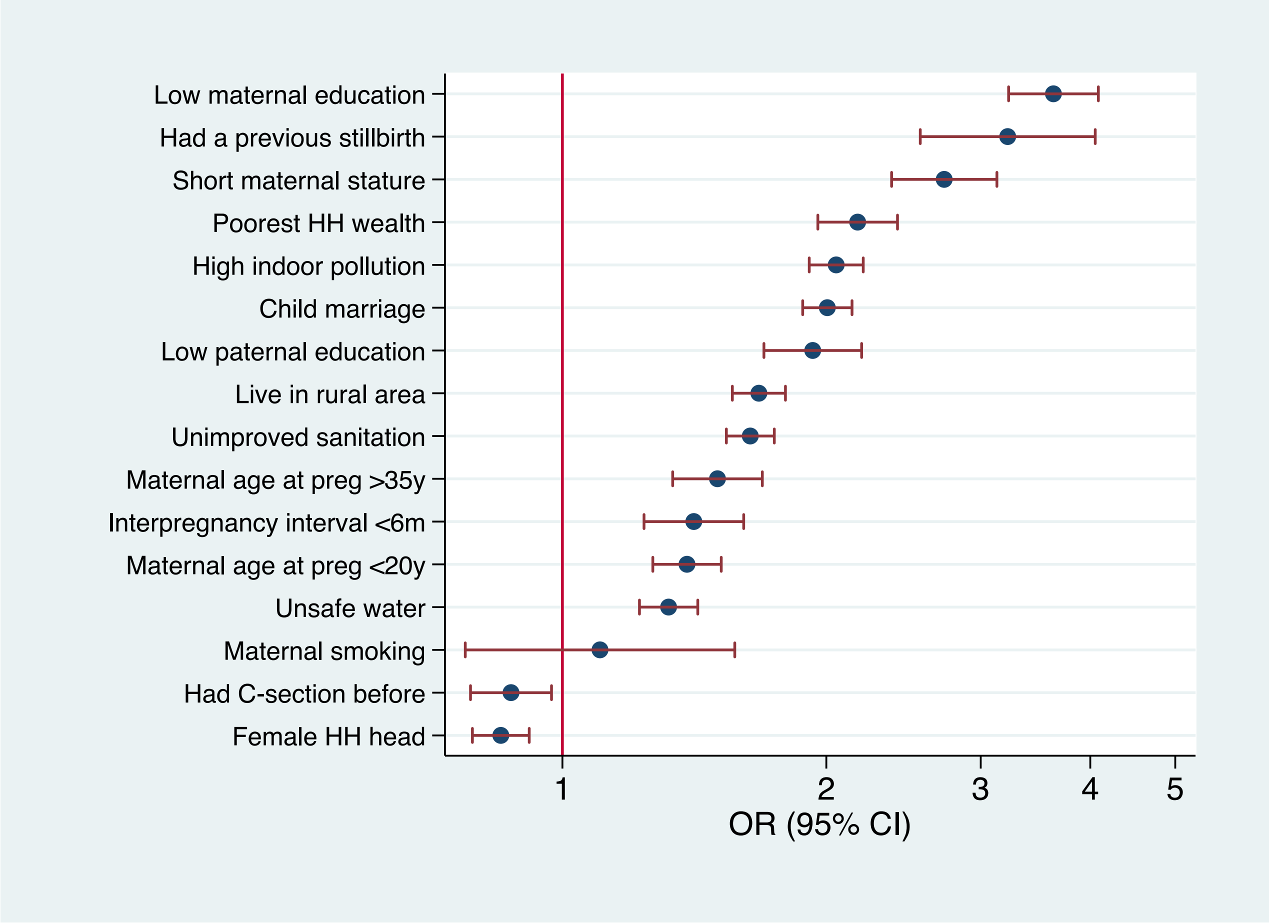

Note:

Low maternal/paternal education – mothers/fathers with no education; short maternal stature - maternal height of less than 145 cm; child marriage - mother younger than 18 years at marriage; poorest HH wealth – household with the poorest wealth status; high indoor pollution – household not using solid fuels for cooking; C-section - caesarean section.

*Maternal age at the survey was adjusted in the models

Appendix Figure 2. Relative ranking of factors associated with stillbirths from mutually-adjusted models, stratified by previous pregnancy history

A) With previous pregnancy history (N=577,772)

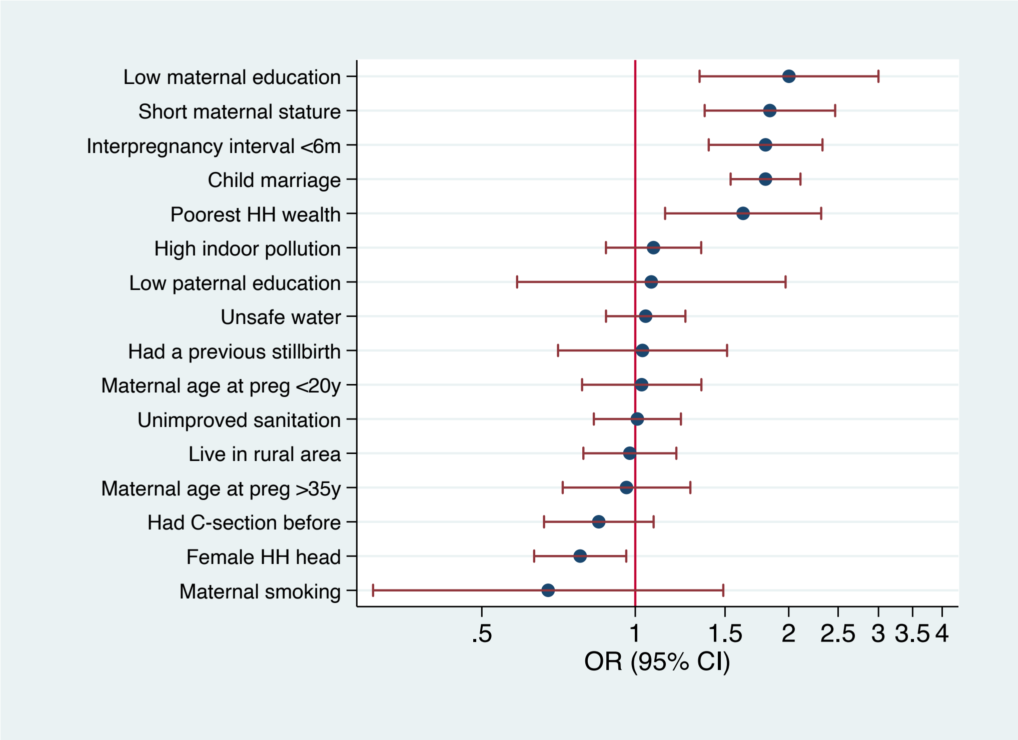

B) Without previous pregnancy history (N=217,870)

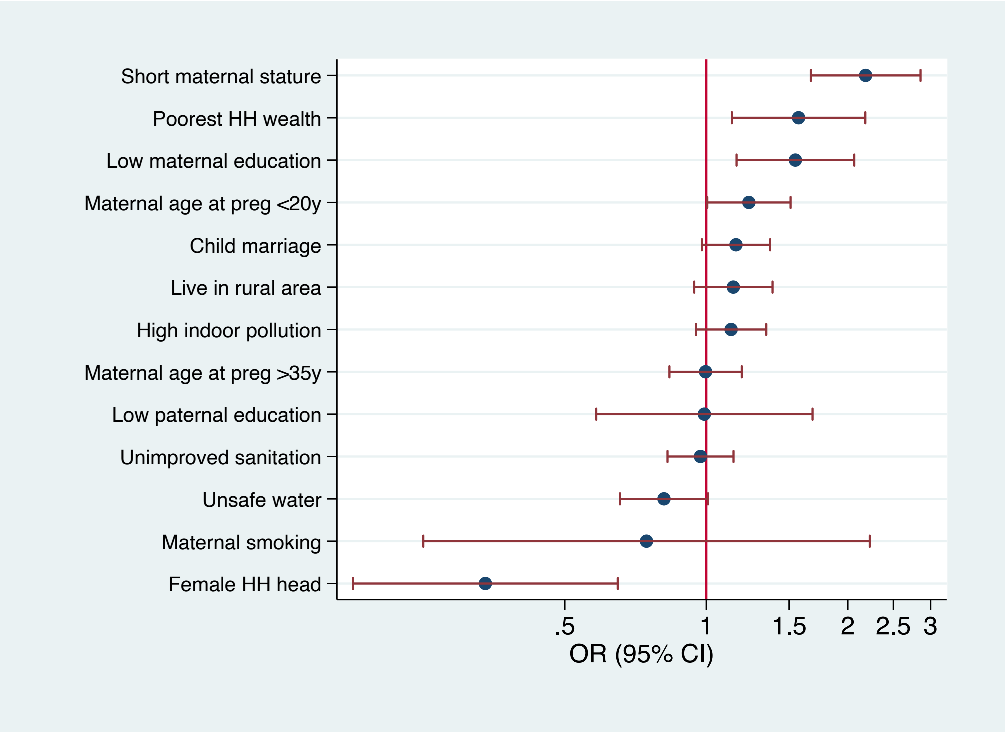

Note:

Low maternal/paternal education – mothers/fathers with no education; short maternal stature - maternal height of less than 145 cm; child marriage - mother younger than 18 years at marriage; poorest HH wealth – household with the poorest wealth status; high indoor pollution – household not using solid fuels for cooking; C-section - caesarean section.

*Maternal age at the survey was adjusted in this model

Appendix Figure 3. Relative ranking of risk factors associated with stillbirths from mutually-adjusted models, stratified by maternal age at pregnancy

A) Maternal age at pregnancy between 15 and 19 (N=138,977)

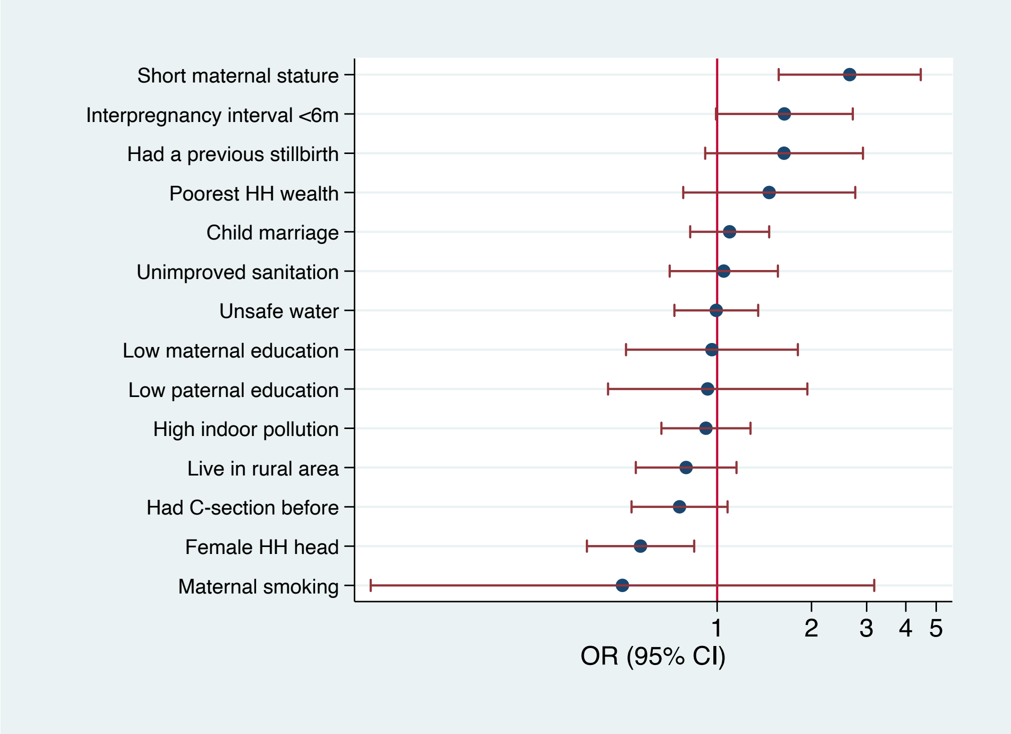

B) Maternal age at pregnancy between 20 and 34 (N=578,555)

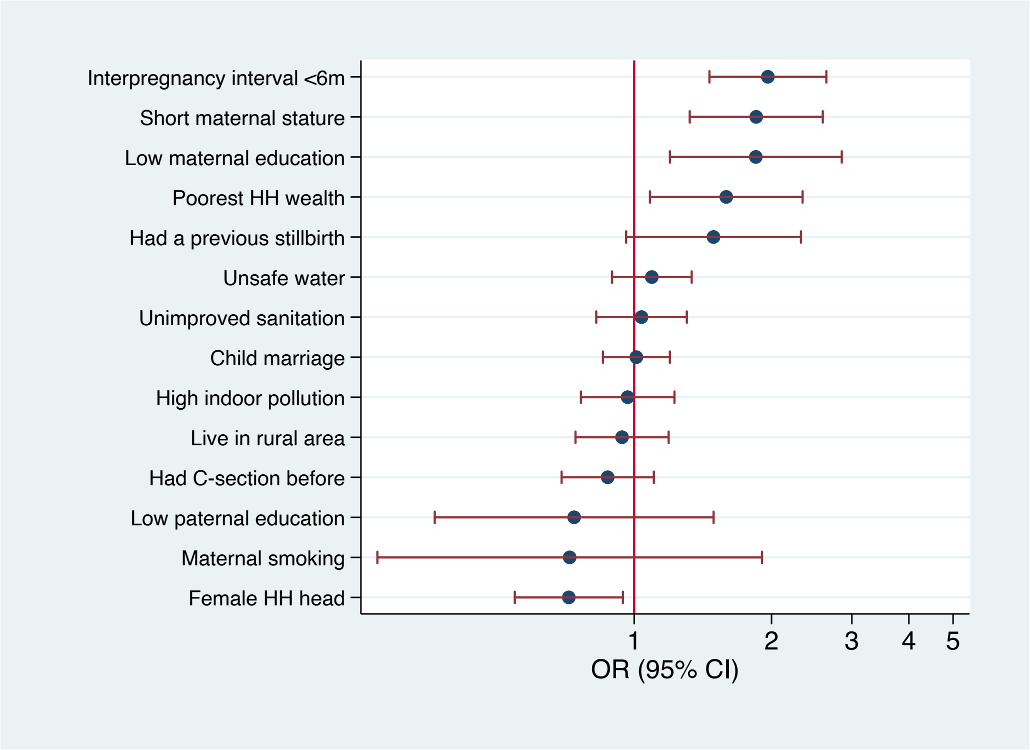

C) Maternal age at pregnancy between 35 and 49 (N=78,110)

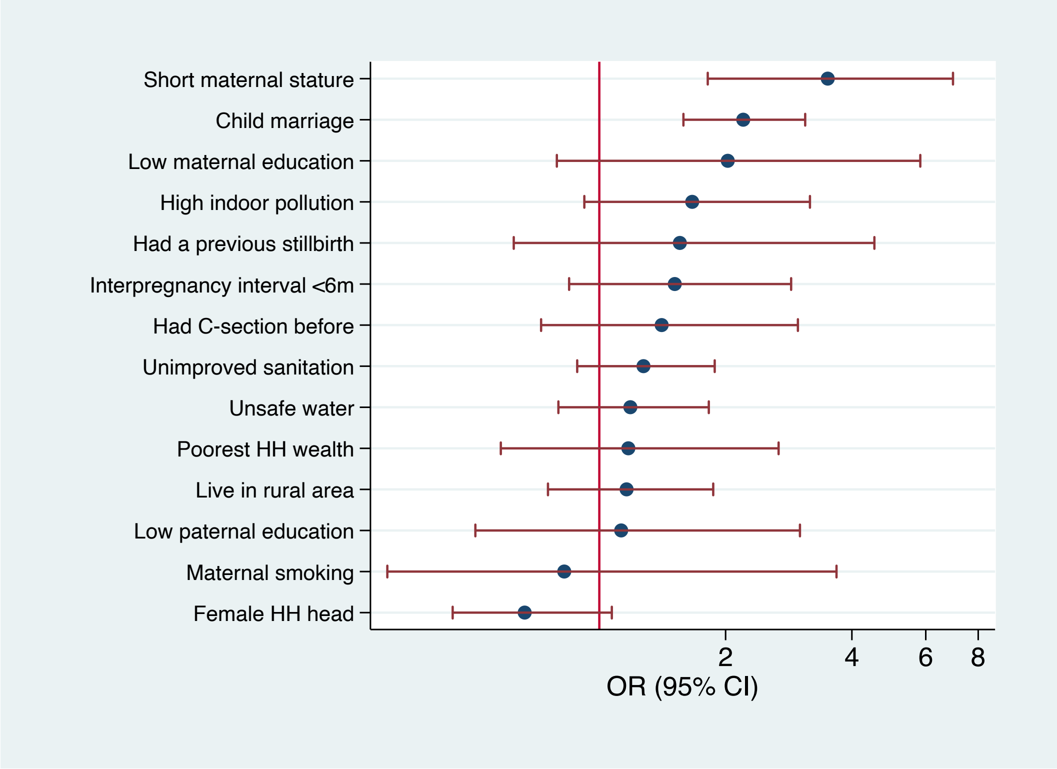

Note:

Low maternal/paternal education – mothers/fathers with no education; short maternal stature - maternal height of less than 145 cm; child marriage - mother younger than 18 years at marriage; poorest HH wealth – household with the poorest wealth status; high indoor pollution – household not using solid fuels for cooking; C-section - caesarean section.

*Maternal age at the survey was adjusted in this model

Appendix Figure 4. Relative ranking of risk factors associated with stillbirths from mutually-adjusted models, adding maternal care indicators (N=272,129)

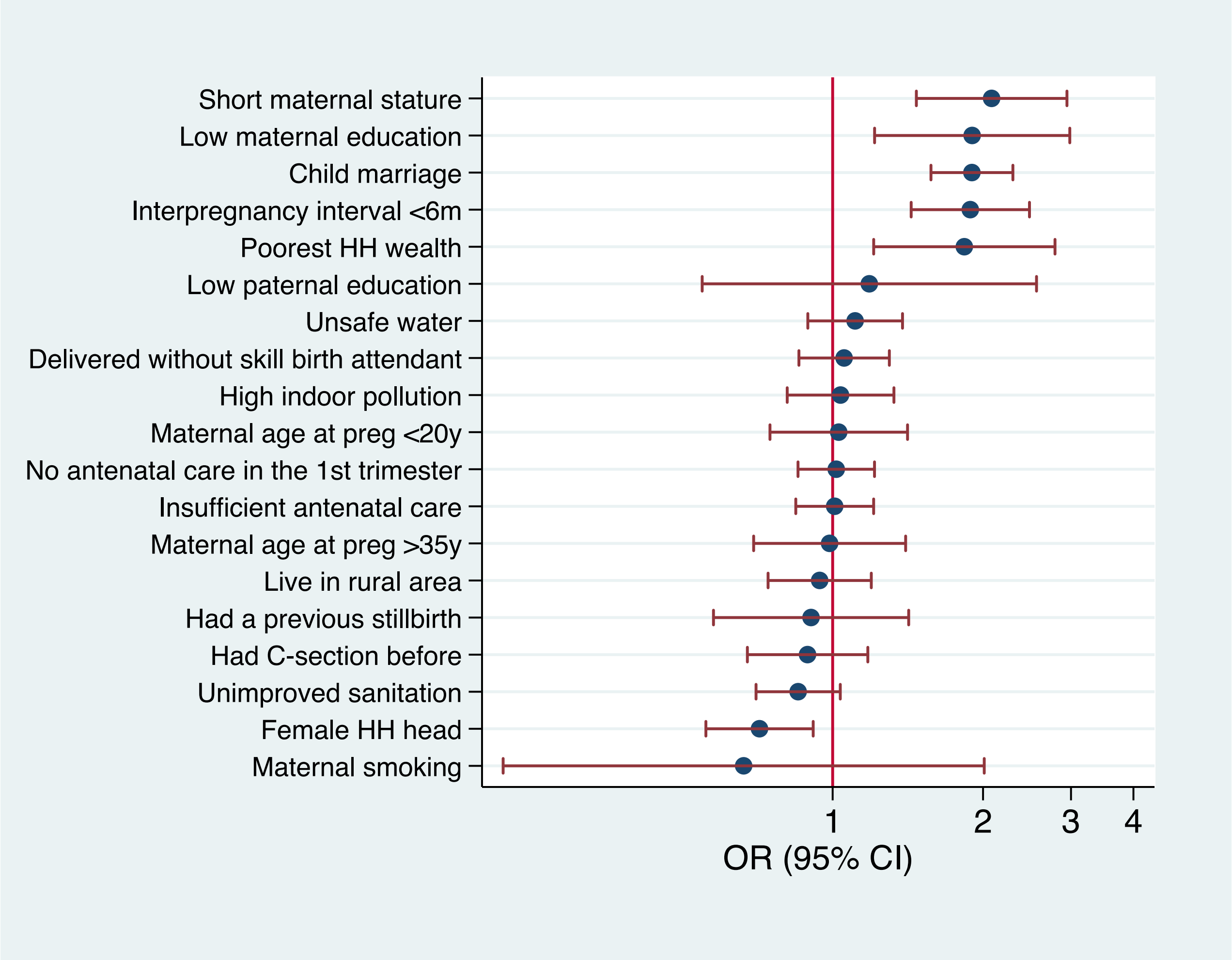

Note:

Low maternal/paternal education – mothers/fathers with no education; short maternal stature - maternal height of less than 145 cm; child marriage - mother younger than 18 years at marriage; poorest HH wealth – household with the poorest wealth status; high indoor pollution – household not using solid fuels for cooking; C-section - caesarean section

*Maternal age at the survey was adjusted in this model

Appendix Figure 5. Relative ranking of risk factors associated with stillbirths from mutually-adjusted models, adding paternal indicators (N=54,248)

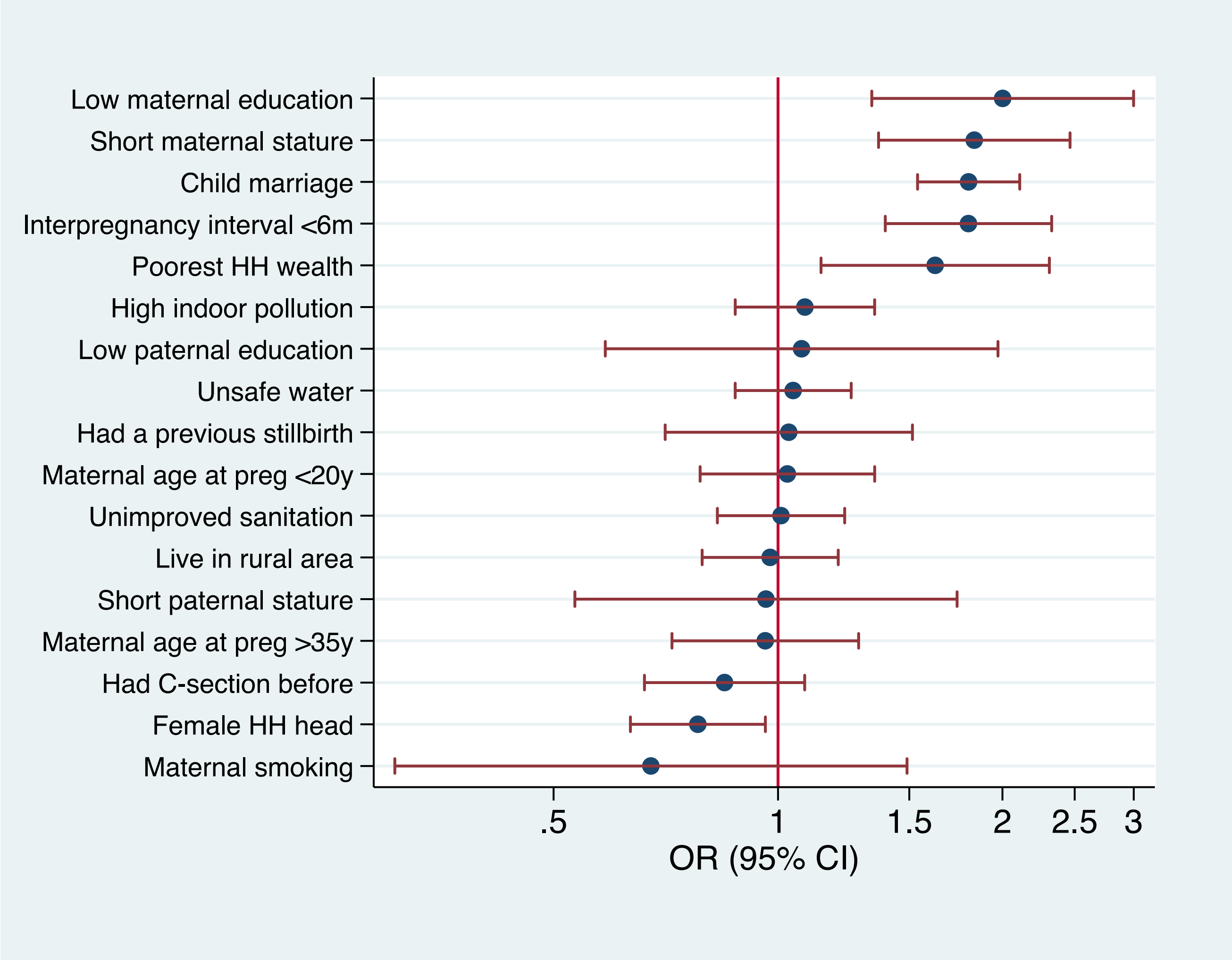

Note:

Low maternal/paternal education – mothers/fathers with no education; short maternal/paternal stature – maternal/paternal height of less than 145/160 cm; child marriage - mother younger than 18 years at marriage; poorest HH wealth – household with the poorest wealth status; high indoor pollution – household not using solid fuels for cooking; C-section - caesarean section.

*Maternal age at the survey was adjusted in this model

Appendix Figure 6. Relative ranking of risk factors associated with stillbirths from mutually-adjusted models, adding maternal weight indicators (N=795,642)

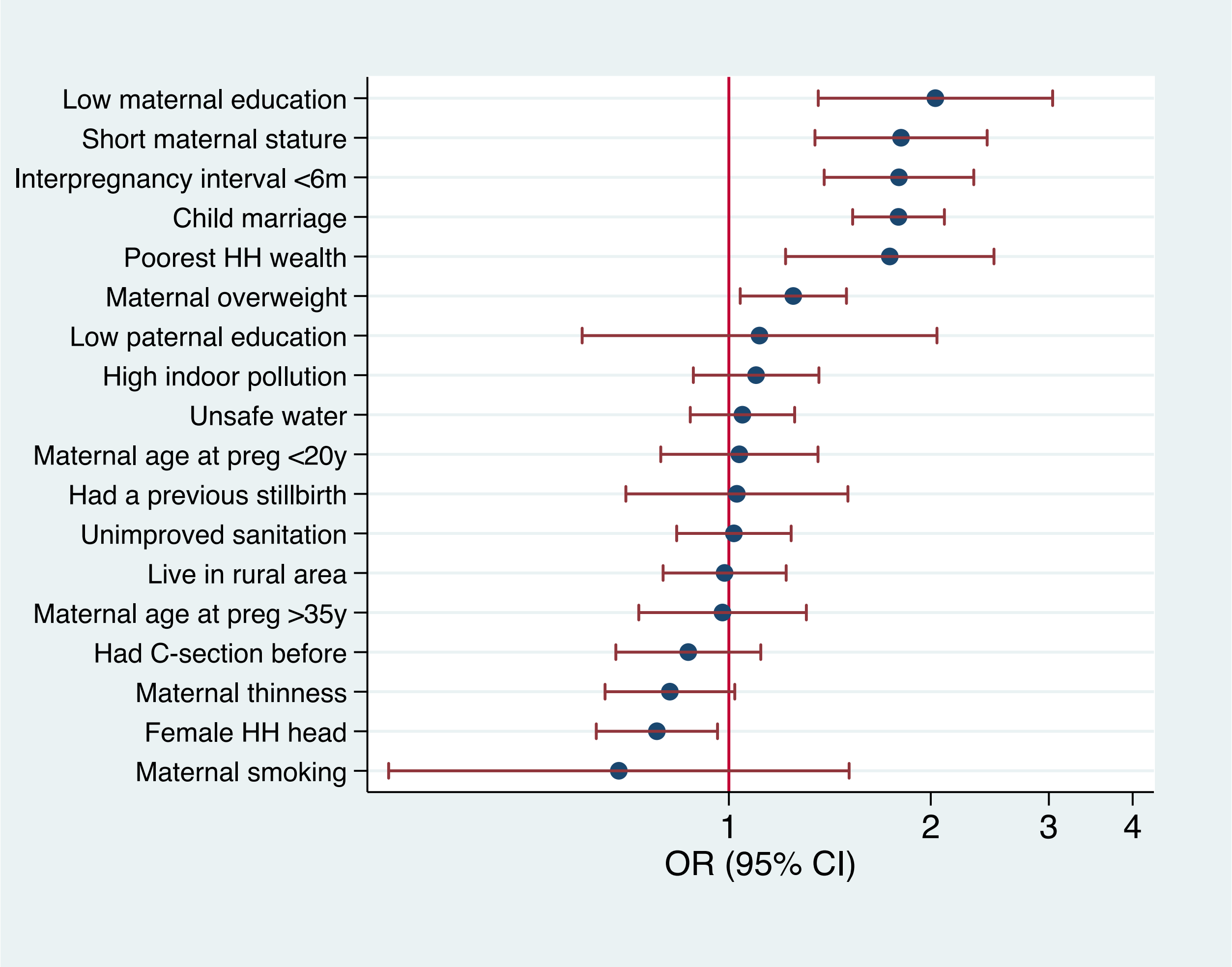

Note:

Low maternal/paternal education – mothers/fathers with no education; short maternal stature - maternal height of less than 145 cm; child marriage - mother younger than 18 years at marriage; poorest HH wealth – household with the poorest wealth status; high indoor pollution – household not using solid fuels for cooking; C-section - caesarean section; maternal thinness: BMI<18.5 kg/m^2; maternal overweight: BMI>25 kg/m^2.

*Maternal age at the survey was adjusted in this model
